# Translation and language validation of the Epworth sleepiness scale for children and adolescents (ESS-CHAD) into Brazilian Portuguese

**DOI:** 10.5935/1984-0063.20220072

**Published:** 2022

**Authors:** Magda Lahorgue Nunes, Felipe Kalil Neto, Marta Hemb, Camila dos Santos El Halal

**Affiliations:** 1 Pontifical Catholic University of Rio Grande do Sul, Brain Institute - Porto Alegre - RS - Brazil; 2 Pontifical Catholic University of Rio Grande do Sul, School of Medicine - Porto Alegre - RS - Brazil

**Keywords:** Children, Adolescents, Sleepiness, Sleep, Validation

## Abstract

**Objective:**

This study aimed to translate the Epworth sleepiness scale for children and adolescents (ESS-CHAD) into Brazilian Portuguese.

**Material and Methods:**

The translation and language validation processes were carried out through translation, back translation, technical review, assessment of verbal comprehension/clarity of the scale by experts (four pediatric neurologists). After they have reached a final version of the ESS-HAD a sample of asymptomatic children and adolescents that were participants in another sleep project were invited to read and complete the questionnaire to evaluate comprehension by the aimed population.

**Results:**

Two independent researchers made the forward translation and it has around 90% of concordance. Minor disagreements were related to the position of adjectives in the sentence. No major difficulties were reported by the 3^rd^ researcher that performed the back translation. After a consensus meeting with the four participants, we have reached a final version of the questionnaire. In the cognitive interviews, the scale was reportedly easy to understand to the 23 respondents. One adolescent suggested clarifying whether question 2 (likelihood of falling asleep watching TV or a video), referred to daytime or nighttime. The total ESS-CHAD score in this asymptomatic sample varied from 0-17, a mean score of 7.08±5.65.

**Discussion:**

The final version of the ESS-CHAD in Brazilian Portuguese was approved by the copyright owners and was well understandable by caregivers and adolescents. More studies are now necessary to use this questionnaire in a larger target population to verify its validity and internal consistency.

## INTRODUCTION

The term excessive daytime sleepiness (EDS) refers to unintended daytime sleep episodes. It is defined as “the tendency to fall asleep at times and under circumstances when the intention and expectation is to remain awake” and is truly different from naps that occur after lying down purposefully. EDS should also be distinguished from other tendencies or aspects of sleep habits, such as being hard to wake in the morning^1,2^.

For the adult population, a variety of laboratory-based methods are available to measure sleepiness and EDS such as the multiple sleep latency test (MSLT) and the maintenance of wakefulness’ test (MWT). Whilst for children there is no consensus on how these symptoms should be evaluated^3^.

In 1991, Johns^4^ proposed a new method for measuring daytime sleepiness, the Epworth sleepiness scale (ESS) and this brief, easy to apply questionnaire, became a tool of worldwide use to evaluate EDS not only on clinical scenarios but also in research. The ESS is an 8-item self-report questionnaire assessing the likelihood of falling asleep in specific everyday situations, thus evaluating an individual’s level of daytime sleepiness. The responses are done on a four-point Likert scale (0-3). The original scale has been translated to Brazilian Portuguese by Bertolazi et al. (2009)^5^ and showed good equivalence with the English version in detecting daytime sleepiness.

The adult ESS is not suitable for young children and even for adolescents. As an example, it should be implied that sleepiness during traveling in a car refers to them as passengers rather than drivers and alcohol questioning is probably not useful for the majority of responders. Although the ESS is subject to copyright (©MW Johns, 1990-1997), many non-authorized adaptations are available, some of which involved minimal changes but others suffered substantial modifications. Under the auspices of copywriter, owners Wang et al.^1^ have adapted the ESS for children and adolescents. They have launched the official version of “Epworth sleepiness scale for children and adolescents” (ESS-CHAD) on 2017. The original options not useful for children such as “sitting quietly without alcohol consumption, driving, and taking part in a meeting” were replaced by “sitting by yourself after lunch”, “sitting as a passenger in a car or a bus”, “sitting in a classroom at school”.

To have reliable and consistent measure of sleepiness in adolescents is important as there are consistent evidences that sleepiness at this age period is linked to issues related to physical and mental health and also poor academic performance^6-12^. Although some other scales are available in Portuguese to measure daytime sleepiness, such as the “the pediatric daytime sleepiness scale (PDSS)”^13,14^, to have another option to evaluate sleepiness in children and adolescents, using a tool that is widely accepted worldwide would also be helpful to clinicians and researchers.

The aim of this article was to translate to Brazilian Portuguese the official version of ESS-CHAD with authorization by the copyright owners.

## MATERIAL AND METHODS

The first step of the process was to ask permission to translation from Mapi Research Trust (copyright owner).

This modified ESS for use in children and adolescents is brief and self-administered. Each question can be scored from 0 (no chance of falling asleep) to 3 (high chance of falling asleep), and a score higher than 10 suggests excessive daytime sleepiness, with a linear correspondence between scores and daytime sleepiness (Table 1).

**Table 1:** Scores of EES-CHAD.

Score	Significance
0-5	Lower normal daytime sleepiness
6-10	Higher normal daytime sleepiness
11-12	Mild excessive daytime sleepiness
13-15	Moderate excessive daytime sleepiness
16-24	Severe excessive daytime sleepiness

The translation of EES-CHAD to Brazilian Portuguese was in agreement with the methodology proposed the copyright owner (Mapi Research Trust) and followed the steps suggested by Acquadro et al. (2004)^15^. Those steps include forward translation, backward translation, review by clinicians, cognitive interviews and international harmonization. Four pediatric neurologists with different expertise on sleep medicine participated in the process, two of them performed the forward translation, the third did the back translation and the 4^th^ coordinated the whole process and solved minor disagreements. In order to verify if the final version was easily understandable by adolescents and caregivers, we have invited participants of another ongoing study to answer the scale, independently. The last step is used only if more than one language is involved, which was not the case of our proposal.

### Agreement and authorization - Ethical aspects

A user license agreement special terms (number 34385) was issued between Mapi Research Trust, the author MLN and the Pontifical Catholic University of Rio Grande do Sul. On April 20^th^, 2021 a permission to start the translation process was issued. On July 19^th^, 2021, through the request 2104360 to ePROVIDE™ the authors were allowed to submit the translation process as an article.

Responders of the cognitive interview were participants of the project “Sleep quality among parents and their children during COVID-19 pandemic”, approved by the institutional ethical committee and registered on *Plataforma Brasil* under the number 30748320.5.0000.5336.

## RESULTS

The complete translation process is detailed on Table 2.

**Table 2:** Translation and Back translation process.

Translation Steps				
**Original**	**Translation 1**	**Translation 2**	**Portuguese version send to Back translation**	**Back translation**
Epworth sleepiness scale for children and adolescents ESS-CHAD	*Escala Epworth de sonolência para crianças e adolescentes (ESS-CRAD)*	*Escala de sonolência de Epworth para crianças e adolescentes*	*Escala de sonolência de Epworth para crianças e adolescentes* ^*^	Epworth sleepiness scale for children and adolescents
Your name	*Seu nome:*	*Nome:*	*Seu nome:*	Your name:
Today’s date	*Data de hoje:*	*Data de hoje:*	*Data de hoje:*	Date:
How old are you?	*Qual sua idade? (anos)*	*Quantos anos você tem? (anos)*	*Quantos anos você tem? (anos)*	How old are you? (years)
Boy? Or Girl? **check** one space	*Menino? ( ) ou Menina ? ( ) preencha um espaço*	*Menino? (_) ou menina? (_) marque um espaço*	*Menino? (_) ou menina? (_) marque um espaço*	Boy? ( ) or girl? ( ) **tick** one spacew
Over the past month, how likely **have you been** to fall asleep while doing the things **that are** described below (activities)?	*No último mês, com que frequência você adormeceu enquanto fez as coisas que são descritas abaixo (atividades)?*	*No último mês, qual a probabilidade de você adormecer enquanto realiza as coisas descritas abaixo (atividades)?*	*No último mês, qual a probabilidade de você adormecer enquanto realiza as coisas descritas abaixo (atividades)?*	Over the last month, how likely **were you** to fall asleep while doing the things described below (activities)?
Even if you haven’t done some of **these** things in the **past** month, try to imagine how they would have affected you.	*Mesmo que você não tenha feito algumas dessas coisas no último mês, tente imaginar como elas afetariam você.*	*Mesmo que você não tenha feito algumas dessas coisas no mês passado, tente imaginar como elas o teriam afetado.*	*Mesmo que você não tenha feito algumas dessas coisas no último mês, tente imaginar como elas afetariam você.*	Even if you haven’t done some of **those** things in the **last** month, try to imagine how they would have affected you.
Use the following scale to choose **one** number that **best** describes what has been happening to you during each activity over the **past** month. Write that number in the box below.	*Use a escala seguinte para selecionar um número que melhor descreve o que tem acontecido em cada atividade com você durante o último mês.*	*Use a seguinte escala para escolher um número que melhor descreve o que tem acontecido com você durante cada atividade no mês passado. Escreva esse número na caixa abaixo.*	*Use a seguinte escala para escolher um número que melhor descreve o que tem acontecido com você durante cada atividade no último mês .* *Escreva esse número na caixa abaixo.*	Use the following scale to choose **the** number that **better** describes what has been happening to you during each activity over the **last** month. Write that number on the box below.
0 = would never fall asleep	*0 = nunca iria dormir*	*0 = nunca iria dormir*	*0 = nunca iria dormir*	0 - Would never fall asleep
1 = **slight** chance of falling asleep	*1 = chance pequena de dormir*	*1 = chance leve de adormecer*	*1 = chance pequena de adormecer*	1 - **Small** chance of sleeping
2 = moderate chance of falling asleep	*2 = chance média de dormir*	*2 = chance moderada de adormecer*	*2 = chance moderada de adormecer*	2 - Moderate chance of falling asleep
3 = high chance of **falling asleep**	*3 = chance grande de dormir*	*3 = chance alta de adormecer*	*3 = chance grande de adormecer*	3 - High chance of **sleeping**
It is important that you answer each question as **best** you can	*É importante que você responda a cada pergunta da melhor forma que puder*	*É importante que você responda cada pergunta o melhor que puder*	*É importante que você responda cada pergunta o melhor que puder*	It is important that you answer each question as **well** as you can
Activities	*Atividades*	*Atividades*	*Atividades*	Activities
Chance of falling asleep (0 - 3)	*Chance de dormir*	*Chance de adormecer*	*Chance de adormecer*	Chance of falling asleep
Sitting and reading	*Sentado e lendo*	*Sentado e lendo*	*Sentado (a) e lendo*	Sitting and reading
Sitting and watching TV or a video	*Sentado e assistindo TV ou vídeo*	*Sentado e assistindo TV ou vídeo*	*Sentado(a) e assistindo TV ou vídeo*	Sitting and watching TV or a video
Sitting in a classroom at school **during** the morning	*Sentado em sala de aula durante a manhã*	*Sentado em uma sala de aula da escola durante a manhã.*	*Sentado em uma sala de aula na escola durante a manhã.*	Sitting in a classroom at school **in the** morning
Sitting and riding in a car or bus for **about** half an hour	*Sentado e andando em um carro ou ônibus por cerca de meia hora*	*Sentado e andando em um carro ou ônibus por cerca de meia hora*	*Sentado(a) e andando em um carro ou ônibus por cerca de meia hora*	Sitting and riding in a car or bus for **around** half an hour
Lying down to rest or nap in the afternoon	*Deitado para descansar ou tirar uma soneca durante a tarde*	*Deitado para descansar ou cochilar à tarde*	*Deitado para descansar ou cochilar à tarde*	Lying down to rest or nap in the afternoon
Sitting and talking to someone	*Sentado e conversando com alguém*	*Sentado e conversando com alguém*	*Sentado (a) e conversando com alguém*	Sitting and talking to someone
Sitting quietly **by yourself** after lunch	*Sentado quieto e sozinho após o almoço*	*Sentado em silêncio sozinho após o almoço*	*Sentado em silêncio sozinho após o almoço*	Sitting quietly **alone** after lunch
Sitting and eating a meal	Sentado e comendo uma refeição	Sentado e comendo uma refeição	Sentado(a) e comendo uma refeição	Sitting and having a meal
Thank you	Obrigado	Obrigada	Obrigado(a)	Thank you

Legends: 100% concordance of translation among the 2 Portuguese versions; 100% concordance among original English version and back translation; **In bold spare words in disagreement between the English version and Backtranslation.**

Notes:

* The adult version of Epworth scale was translated to Brazilian Portuguese as: “Escala de Sonolência de Epworth em português do Brasil” (ESE-BR).

**1. Forward translation:** two pediatric neurologists fluent in English made the forward translation. Subsequently, both translations were discussed between the authors until agreement on the most adequate Portuguese version. The translations had around 90% of concordance and minor disagreements were related to position of adjectives in the sentence. In this step a senior pediatric neurologist conducted the discussion and decided in case of disagreement the for the best version.

**2. Backward translation:** the back translation was performed by a Brazilian Pediatric Neurologist fluent in English. According to the ratings proposed by Acquadro et al. (2004)^15^ to describe the difficulty of translating the wording of the source document, the level of difficult found by the translator was scored as 1 (no difficulty: no problems expected in developing a rendering that faithfully captures a concept that is equivalent to the source text).

**2.1. Review by clinicians:** all authors have independently reviewed the final Portuguese version. Finally, in a consensus meeting, the most accurate translation was chosen, considering the best conceptual, semantic, and cultural equivalences.

**2.2. Cognitive interviews:** to evaluate comprehension by the aimed population 23 asymptomatic children and adolescents were asked to read and complete the questionnaire. In one case, the mother of a four-year-old child completed the scale. The scale was reportedly easy to understand. One adolescent suggested to clarify whether question 2 (likelihood of falling asleep watching TV or a video), referred to daytime or nighttime. Table 3 shows the results of the questionnaires answered for the cognitive interviews. Participants’ age varied from 4 to 15 years, mean age of boys was 10 years (median 11-12 years) and for girls 11 years (median 11 years). There was a predominance of girls responders’ (65.2%). In all situations proposed the chance of falling asleep varied from 0 to 3, except on the last one (chance of falling asleep while eating a meal) where all responders answered never. The mean score for each situation was: sitting and reading = 1, sitting and watching TV or a video = 1.08, sitting in a classroom at school during the morning = 0.78, sitting and riding in a car or bus for about half an hour = 1.73, lying down to rest or nap in the afternoon = 1.5, sitting and talking to someone = 0.04, sitting quietly by yourself after lunch = 1.03, sitting and eating a meal = 0. The total ESS-CHAD score varied from 0-17, mean score 7.08±5.65.

**Table 3:** Description of results of the Cognitive interview.

Age (y)	Sex	Sitting and reading	Sitting and Watching TV/video	Sitting in classroom at school during the morning	Sitting and riding car/bus for about half an hour	Lying down to rest or nap in the afternoon	Sitting and talking to someone	Sitting quietly by yourself after lunch	Sitting and eating a meal	EES-CHAD total score	Daytime sleepiness
7	M	1	2	2	3	3	0	1	0	10	HNDS
13	F	1	1	1	2	3	0	1	0	9	HNDS
9	F	1	0	3	2	3	0	2	0	11	MEDS
8	F	2	0	1	3	3	0	3	0	12	MEDS
10	F	1	1	0	0	0	0	0	0	2	LNDS
14	M	0	1	1	2	3	0	2	0	9	HNDS
15	F	0	2	1	2	3	0	1	0	9	HNDS
11	M	1	1	0	3	1	0	1	0	7	HNDS
8	F	0	3	0	2	0	0	0	0	5	LNDS
15	F	1	3	1	3	3	0	3	0	14	MOEDS
13	F	3	1	1	2	0	0	2	0	9	HNDS
^*^4	M	1	0	1	3	0	0	0	0	5	LNDS
15	F	1	3	0	0	2	0	1	0	7	HNDS
7	M	1	0	2	0	0	0	0	0	3	LNDS
13	F	2	1	0	3	3	0	1	0	10	HNDS
11	F	1	1	0	2	0	0	0	0	4	LNDS
14	F	2	1	0	1	3	0	1	0	8	HNDS
13	M	0	1	0	0	1	0	0	0	2	LNDS
12	M	1	0	0	1	1	0	1	0	4	LNDS
15	M	3	2	3	3	3	1	2	0	17	SEDS
11	F	0	0	0	0	0	0	0	0	0	LNDS
7	F	0	1	1	2	0	0	0	0	4	LNDS
10	F	0	0	0	1	0	0	1	0	2	LNDS

Notes:

* The questionnaire was answered by the mother; LNDS = Lower normal daytime sleepiness; HNDS = Higher normal daytime sleepiness, MEDS = Mild excessive daytime sleepiness; MOEDS = Moderate excessive daytime sleepiness; SEDS = Severe excessive daytime sleepiness.

The final version of the ESS CHAD scale translated to Brazilian Portuguese was sent to Mapi Research Trust, was approved and can be obtained at (https://eprovide.mapi-trust.org/instruments/epworth-sleepiness-scale-child-adolescent^16^).

## DISCUSSION

In this study, we have translated to Brazilian Portuguese the official version of the ESS-CHAD. All steps recommended by Mapi Research Trust (copyright owners) were followed. It is worthy to have a Brazilian Portuguese version of this instrument as this questionnaire is based in the well-established and well-validated adult ESS. It is fairly known that many questionnaires are available to evaluate sleep in pediatric ages^13,14,17^. However, this modified version, with questions very similar to those of the adult ESS, might be useful not only to screen patients but also for clinical research.

An objective way to measure excessive sleepiness during the day is useful as previous studies have pointed to an increase of sleep disorders in adolescents, where a sleep phase delay is characteristic and this symptom could indicate a shorter sleep duration or unfavorable sleep quality. In children and adolescents, it might be associated, as consequences, with learning and behavioral problems and attention disorders^10-12,18-20^. Further, sleepiness can also be a pathway linking race and socioeconomic status with worse academic and cognitive outcomes in middle childhood^21^.

One advantage of the ESS-CHAD over the PDSS is the fact that the first has a cutoff point that differentiates normal daytime somnolence to abnormal, whilst PDSS shows a measure of association, being higher scores related to more diurnal somnolence^6^.

The psychometric analysis of the ESS-CHAD was previously described by Janssen et al. (2017)^22^. They found that this scale is a reliable and internally valid measure of daytime sleepiness for adolescents with an age range between 12-18 years old.

During the COVID-19 pandemic, many studies had evaluated sleep in children/adolescents^11,12,23^. Bruni et al. (2021)^11^ found a big delay in the sleep-wake schedule in all age groups as well as an increase of sleep disturbances, in a large cohort of Italian children and adolescents. A similar study developed in southern Brazil including dyads of parents and children observed excessive daytime somnolence in almost 10% of the group with 4-12 years of age^23^. The increase in sleep disorders during the pandemics was observed worldwide and instruments to screen the pediatric population are very useful to establish an adequate clinical approach during the follow up.

The final version of the ESS-CHAD in Brazilian Portuguese was approved by the copyright owners and was well understandable by caregivers and adolescents that participated in this project. More studies are now necessary to use this questionnaire in a larger target population to verify its validity and internal consistency.
